# HLA-genotyping and the historical naming process of an old disorder

**DOI:** 10.1590/1677-5449.20220036

**Published:** 2022-05-25

**Authors:** Cem Evereklioglu

**Affiliations:** 1 Department of Ophthalmology, Division of Uvea-Behçet Unit, Erciyes University Medical Faculty, Kayseri, Turkey.

Dear Editor,

I read the article by Metzger et al. entitled “*Endovascular treatment of aortic saccular aneurysms associated with Adamantiades-Behçet disease*” (BD, 2021). They reported a 49-year-old man with an abdominal aorta aneurysm treated with an inferior vena cava filter and endovascular repair. I thank the authors for their efforts. However, I have 2 questions to be clarified:

1) They stated that BD has associations with HLA-B51 and HLA-B27. However, BD is strongly associated with HLA-B51,^1^^,^^2^ but weakly with HLA-B27^3^ and there is no study linking BD strongly with HLA-B27. Indeed, an HLA-B27-related non-infectious entity is associated with ankylosing spondylitis uveitis.^4^^,^^5^ I wonder whether the authors performed HLA analyses or did they demonstrate any link with HLA-B27 or HLA-B51?

2) This disorder was called by the authors “Adamantiades-Behçet”. Investigators before Hulusi Behçet reported several Behçet findings since Hippocrates,^6^ such as Planner and Remenovsky (1922)^7^ and Pils (1925).^8^ However, none indicated a novel disease with the “classical-triad”. Adamantiades concluded that “hypopyon iritis” (not classical-triad) constitutes a distinct entity, which can occur in many disorders.^9^ Hulusi Behçet was the first to realize and group dermato-ophthalmological findings into one disease (triple-symptom-complex; 1937-1940).^10^^-^12 Metzger et al. used the “International Study Group Criteria for BD” (not Adamantiades-Behçet). The authors should respect the name of an established old disease and no-one should try to change it. Therefore, the authors should address the reason for preferring the eponym Adamantiades-Behçet. Why should only Dr. Adamantiades be honored? PubMed reveals 13,315 Behçet articles (1946-2022), and 175 Adamantiades-Behçet (1970-2022). Similarly, they used 17 articles in the reference and only one paper stated it as “Adamantiades-Behçet”. It is the authors’ responsibility to use the correct, accepted, and established international eponym because editors may not realize such a historical naming process. The “Jornal Vascular Brasileiro” published 5 articles, all using Behçet without exception. Moreover, the American BD Association, International BD Society, and Japan BD Committee all call the disease “Behçet” to honor the first describer of the “triple-symptom-complex”. Furthermore, textbooks of dermatology, rheumatology, and ophthalmology, and international congresses call this entity BD, not Adamantiades-Behçet. Adamantiades himself named the syndrome “Behçet”.^13^ I think it is time to abandon efforts to change the name of an old syndrome.
