# Supplementary material for: HLA-genotyping and the historical naming process of an old disorder
Source: J Vasc Bras. 2022 May 25;21:e20220036. doi: 10.1590/1677-5449.20220036 (PMC9136685; doi:10.1590/1677-5449.20220036)
Supplement: Related texts

# Reply: RESPONSE LETTER

**Authors:** Patrick Bastos Metzger, Kamilla Rosales Costa, Simone Lessa Metzger, Leonardo Cortizo de Almeida

**Affiliations:** 1 Escola Bahiana de Medicina e Saúde Pública (EBMSP), Salvador, BA, Brasil.; 2 Universidade Salvador (UNIFACS), Salvador, BA, Brasil.; 3 Hospital Santo Antônio, Obra Sociais Irmã Dulce (OSID), Salvador, BA, Brasil.; 4 Hospital Geral Roberto Santos (HGRS), Salvador, BA, Brasil.

Dear Editor,

We read the aforementioned letter to the editor about the article we published entitled: Endovascular treatment of aortic saccular aneurysms associated with Adamantiades-Behçet disease^1^. We thank the author for reading and for his well-founded questions. Below are the answers to clarify the questions:

1- In this study we did not perform HLA analysis. A large number of studies have shown a link between the disease and HLA-B51 and HLA-B27 antigen.^2^^-^5 It has been shown that susceptibility to Adamantiades-Behçet disease or Behçet disease is strongly associated with the presence of HLA-B51 cells and the frequency within families has been shown to be up to 15%.^2^ Environmental factors, such as infectious agents, have also been implicated in this pathogenesis.^2^ However, the correlation with HLA-B27 is significant, although not strong, as demonstrated in the recent 2019 meta-analysis published by Khabbazi et al.,^6^ in which they determined that the prevalence of “HLA-B27” in patients with Behçet disease compared with controls was 1.55 (95%CI 1.01-2.38), P = 0.04.
2- In 1930, Benediktos Adamantiades (1875-1962), Greek ophthalmologist from Prussa - Asia minor, presented a lecture with the title “A case of relapsing iritis with hypopyon”, describing a 20-year-old male patient with the three cardinal signs of the disease. The disease had begun at the age of 18 with edema and ulcerations of the left leg diagnosed as thrombophlebitis. Over the following 2 years (1928-30), the patient developed recurrent iritis with hypopyon in both eyes that led to blindness and atrophy of the optic nerve, scrotal ulcers healing with scars, oral aphthous ulcers, and sterile arthritis of both knees. The last three signs were recurrent. Bacterial cultures of knee and anterior eye chamber punctures were sterile and inoculation experiments in animals were negative, whereas staphylococci grew in cultures from scrotal ulcers and a tonsillar abscess. The lecture was published in the Proceedings of the Medical Society of Athens^7^ in the same year and in the French journal Annales d'Oculistique in the following year.^8^ Adamantiades identified the genital ulcers, the arthritis, and the ocular signs in conjunction as signs of a single disease. In 1946, Adamantiades reported two further patients and defined thrombophlebitis as a fourth cardinal sign of the disease.^9^ Later, he presented the first classification of the disease by describing the ocular, mucocutaneous, and systemic forms in a review article.^10^ He pointed out that the disease can occur for years as a monosymptomatic or oligosymptomatic disorder and that eye involvement and severe prognosis are more common in men than in women. In this paper he also proposed the first diagnostic criteria.

On May 11, 1937, Hulsi Behçet (1889-1948) presented a 34-year-old female patient with recurrent oral aphthous ulcers, genital ulcers, and ocular lesions for 7 years at the meeting of the Dermatological Association of Istanbul. This patient’s case was published by Behçet the same year, together with a 40-year-old old man with a disease history of over 20 years.^11^ The microscopic Giemsa preparation from an oral ulcer of the first patient showed structures whose size corresponded to smallpox elementary bodies and therefore Behçet initiated the hypothesis of the viral etiology of the syndrome.^12^ Over the following 3 years he published articles on five further patients in different languages.^12^^-^16 In these publications he added periodontitis, jaw cysts, acneiform skin lesions, erythema nodosum, and arthralgia to the so-called “triple symptom complex”. He was convinced of the autonomy of this multisymptomatic illness and of its viral etiology and finally drew the attention of the scientific community to this puzzling disease.

The first description of the disease was neither by Adamantiades nor by Behçet. Hippokrates of Kos (460-377 BC) had already described an illness whose manifestations fit very well with the cardinal signs of Adamantiades-Behçet’s disease in the fifth century before Christ in his third “Epidemion” book.^17^

The term “Adamantiades-Behçet’s disease” honors both first describers of the several manifestations constituting an autonomous disease in modern times.

The intention of our article was not to discuss the merits of the nomenclature, nor to be little any of the authors who collaborated in recognition of the disease. We recognize as positive all contributions in the process of describing this old syndrome. Therefore, the objective of the article was to report and describe the interventionist approach to an inflammatory disorder with aortic involvement that is not prevalent.

We are glad that our article has generated scientific discussion.

## References

[1] Metzger PB, Costa KR, Metzger SL, Almeida LC (2021). Endovascular treatment of aortic saccular aneurysms associated with Adamantiades-Behçet disease. J Vasc Bras.

[2] Sakane T, Takeno M, Suzuki N, Inaba G (1999). Behçet’s disease. N Engl J Med.

[3] Gürler A, Boyvat A, Türsen U (1997). Clinical manifestations of Behçet’s disease: an analysis of 2147 patients. Yonsei Med J.

[4] Jorizzo JL (1986). Behçet’s disease. An update based on the 1985 International Conference in London. Arch Dermatol.

[5] Chajek T, Fainaru M (1975). Behçet’s disease. Report of 41 cases and a review of the literature. Medicine.

[6] Khabbazi A, Vahedi L, Ghojazadeh M, Pashazadeh F, Khameneh A (2019). Association of HLA-B27 and Behcet’s disease: a systematic review and meta-analysis. Auto Immun Highlights.

[7] Adamantiades B (1930). A case of recurrent hypopyon iritis. Proc Med Soc Athens..

[8] Adamantiades B (1946). Sur un cas d’iritis a hypopion recidivant. Ann Ocul.

[9] Adamantiades B (1953). La thrombophlebite comme quatrieme symptome de l’iritis recidivante a hypopyon. Ann Ocul.

[10] Adamantiadis B (1953). Le symptome complexe de l’uvéite récidivante à hypopyon. Ann Ocul.

[11] Behçet H (1937). Über rezidivierende, Aphthöse, durch ein Virus verursachte Geschwüre am Mund, am Auge und an den Genitalien. Dermatol Wochenschr.

[12] Behçet H (1938). Kurze Mitteilung uber Fokalsepsis mit aphthosen Erscheinungen an Mund, Genitalien und Veranderungen an den Augen, als wahrscheinliche Folge einer durch Virus bedingten Allgemeininfektion. Dermatol Wochenschr.

[13] Behçet H (1938). Considerations sur le lesions aphteuses de la bouche et des parties genitales ainsi que sur les manifestations oculaires d’origine probablement parasitaire et observations concernant leur foyer d’infection. Bull Soc Fr Dermatol Syphiligr.

[14] Behçet H (1939). A propos d’une entite morbide due probablement a un virus special donnant lieu a une affection generalise se manifestant par poussees recidivantes en trois regions principales et occasionnant en particulier des iritis repetes. Bull Soc Fr Dermatol Syphiligr.

[15] Behçet H (1939). Einige Bemerkungen zu meinen Beobachtungen über den Tri- Symptomenkomplex. Med Welt.

[16] Behçet H (1940). Some observations on the clinical picture of the so-called triple symptom complex. Dermatologica.

[17] Zouboulis CC, Keitel W (2002). A historical review of early descriptions of Adamantiades-Behçet’s disease. J Invest Dermatol.
